# Treatment of Teeth with Dead or Dying Pulps, and Also of Alveolar Abscess

**Published:** 1881-11

**Authors:** H. H. Townsend

**Affiliations:** PONTIAC.


### ARTICLE II.

### *Treatment of Teeth with Dead or Dying Pulps; also Treatment of Alveolar Abscess.*

BY DR. H. H. TOWNSEND, OF PONTIAC.

(Read before the Illinois State Dental Society, May 10, 1881.)

*Mr. President and Gentlemen.*—The great number of teeth that are now restored to comfort and usefulness, after disease or death of the pulp, or the pericementum has become inflamed or abscessed, which but a few years ago were lost, with little or no effort being made for their preservation, is certainly very gratifying evidence of progress in this direction. That there are still some obstinate and intractable cases which, despite the most patient and persistent treatment, have thus far proven incurable, while not so gratifying, is evidence that something yet remains to be accomplished. That the success attained by the majority of the profession in the treatment of alveolar abscess is largely due to the essays and discussions of the dental societies, I think will be admitted, and it is with the *hope* that by again debating this question, and comparing ideas and experiences, some new light may be afforded us, and some means and methods suggested which will enable us to treat successfully some of the now incurable cases, that I consented to write upon this subject. An abscess is said to be a collection of pus in a circumscribed cavity. Alveolus is defined as a socket. Hence a collection of pus in the socket of a tooth is an alveolar abscess.

The first symptons of this affection are a slight uneasiness in the tooth, an occasional slight pain, and a little tenderness felt upon pressure. As the disease progresses, the severity of the symptoms increase to an acute pain, elongation and looseness of the tooth, which is so sore that the slightest pressure is unbearable. The gum over the affected tooth becomes swollen, dark red, and very painful. When pus has pierced the bone, fluctuation is easily felt by gently pressing upon the gum with the finger. In some cases there is considerable constitutional disturbance. The tongue is thickly coated; breath quite offensive; skin hot and dry; pulse full and bounding; bowels constipated; and in short, a real inflammatory fever is developed; the formation of pus being announced by a distinct chill. It is interesting to note the great difference in the severity of the symptoms in different cases during the formation of abscesses; frequently a slight soreness and occasional pain being all that is noticed by the patient until pus is discharged through the gum.

The principal causes of this disease are dead or dying pulps confined within the pulp chambers of the teeth; dead or partially dead roots, which is making an effort to expel; and mechanical injuries. To which should be added as occasional causes: the imperfect removal of pulps; imperfectly filled canals; filling the apical portion of canals with cotton; and small canals left untouched. The severity of the disease is modified by the diathesis and health of the patients.

Pathologically, an alveolar abscess is quite similar to a whitlow or felon upon the finger, the difference is principally that of location, both being an inflammation and suppuration of a periosteal tssue.

A brief allusion to the pathology of inflammation may not be out of place, inasmuch as a knowledge of these phenomena is necessary to intelligent treatment. It will be remembered that when an irritant is applied to a healthy tissue, a contraction of the blood vessels first takes place, followed by a corresponding dilatation—an increased flow of blood to the part; an adhering of the red blood corpuscles to the walls of the vessels, and also to each other until they become impacted, and the congestion results in complete stagnation—the dilatation being due to muscular exhaustion of the contractile coats of the vessels. If by any means this congestion can be relieved, the accumulated corpuscles sent off into the current, some stimulant afforded the exhausted coats of the vessels, and the circulation re-established—pus will not form, and the inflammation terminates in resolution. If, however, the congestion goes on to complete stagnation, suppuration must take place, and an abscess is the result. The length of time that an inflammation may exist, before terminating either by resolution or suppuration, varies as greatly as does the severity of the symptoms in different cases. Sometimes an abscess is formed only after weeks of inflammation, and again but few hours are required for the formation of pus.

The source of pus, the precise manner of its formation, of what it is composed, and in what respect it differs from the white blood corpuscles, are questions not yet definitely settled. Practically, with regard to the treatment of alveolar abscess, these are questions which concern us less than that of the *absorption of pus*.

Hamilton's Surgery, page 38, says upon this point that, "It is now pretty generally admitted that pus corpuscles, as such, are never absorbed ; but that, after undergoing certain changes presently to be described, all the elements composing pus may return to the circulation ; yet examples of the spontaneous disappearance of purulent collection by disintegration and absorption, are certainly very rare. Its absorption is effected in the following manner: First, the serum and salts constituting the most fluid parts are removed: second, the pus corpuscles undergo fatty degeneration and disintegration; finally, the pus corpuscles, thus metamorphosed and fluidified, are absorbed, also. In other cases, the serum alone is absorbed, and the salts, with the dessicated pns corpuscles, remain. The pus corpuscles, when thus separated from the serum, dried and aggregated, forming a cheesy mass resembling very much tubercular deposits; the salts when not absorbed remaining as cretaceous layers or nodules."

The treatment of alveolar abscess will depend upon the cause, and the condition in which we find it. If occasioned by a dead root, which nature is trying to expel, the extraction of the root will usually be all the treatment required. If from a blow upon the tooth, or other mechanical injuries, the pulp is still alive, and suppuration has not taken place, the infiammation may be arrested by preventing occlusion with the antagonizing tooth, and the frequent application to the gum around the affected tooth of the tinctures of aconite and iodine, two parts of the former to one of the latter. If pus has already formed, and the pulp still retains its vitality, every effort should be made to arrest the progress of the disease, as the pulp cannot long remain in a state of health if suppuration is allowed to go on; particularly if the sac is near the apex of the root. Here we have a case analagous to the whitlow or felon referred to: suppuration of a periosteal membrane, where the cause is removed. A felon freely opened as soon as pus is formed, usually requires little or no other treatment. The same is true of an abcessed tooth caused by mechanical injury, where the pulp is not diseased. It is rather heroic practice to cut through the gum and alveolus, but I think it is justifiable in such cases. I should, in addition to the opening, paint the gum with aconite and iodine, as there is always some congestion surrounding the suppurating tissue. Where an abscess is caused by a dead or dying pulp, we must first remove the cause. It frequently happens, however, that owing to the extreme soreness of the tooth, and the nervous condition of the patient, it is impossible to do more at the first sitting than to open the pulp chamber and afford vent to the pent up gasses, deferring extirpation and cleansing the root canals until the soreness subsides, which it usually does in a few days, especially if the aconite and iodine is applied to the gums. If the pulp is still too sensitive to admit of extirpation, I should devitalize it with arsenic, using for the purpose not more than 1-250 part of a grain, combined with from 1-32 to 1-16 of a grain of morphia with sufficient creosote to moisten the pellet of cotton, covering in all cases with a temporary filling of gutta percha. It is important that the surplus creosote should be absorbed before introducing the temporary filling, as otherwise it might be forced out, carrying with it some of the arsenic. Accidents have been caused in this way. The length of time which arsenic should be allowed to remain in a tooth varies in different cases. I think, however, it may safely be left until the pulp is entirely devitalized; this varying from a few hours to several weeks. As the last portions of the pulp die, some soreness is usually felt, which generally disappears in a day or two. No further irritation is experienced until mephitic gasses are formed, when extirpation should be no longer delayed. Perhaps the best time for the removal of the pulp is when it is entirely dead and sloughing has taken place, as it then produces no irritation, is painlese, and more easily accomplished than at any other time. I know of no way of determining just when this has taken place, except by testing. Usually, I think it occurs soon after the soreness subsides, occasioned by death of the apical portions of the pulp. I certainly would not advise the removal of a pulp when only partially devitalized, as it is a very painful operation to the patient, and is liable to produce inflammation of the tissue beyond the foramen, which is often very difficult to control.

It is not difficult to say that the pulps should be entirely removed, the canals thoroughly cleansed and perfectly filled; but to state just how this is to be accomplished is not easy—will say, however, that for the successful removal of the pulp three things, at least, I regard as highly essential, viz.: the rubber dam, good light, and very delicate instruments, either entirely soft, or with an excellent spring temper. I wonder that any one should attempt the extirpation of a dental pulp, at the present day, without the aid of the rubber dam; yet I know it is the common practice of many operators. It is true that in a dry mouth the anterior teeth of the upper jaw can be kept tolerably dry with a napkin; but, all things considered, I doubt if as successful operations can be performed in any case without the rubber as with it. In the first place, then, I always apply the rubber dam, and open the cavity sufficiently to thoroughly excavate the decayed dentine; also, open the pulp chamber, so that every part of it can be distinctly seen, with the aid of a mirror. Then, with a warm air cavity drier, reduce the cavity to as nearly absolute dryness as possible; this bleaches the dentine, affording a much better light in the cavity. I then place my patient in the best light practicable, and here let me say that I think the want of a good light is one cause, at least, of many failures in removing pulps and treating roots, as well as any other dental operations. We *must* have light to see what we are doing. At first I operated by a north window, but in cloudy weather my light was not satisfactory; next, tried a west—this, in dark days, was good only in the afternoon; I then tried a south, which I liked much better than either of the others. For the past five years have operated by a south and west. Each window is supplied with two curtains, a green and a white, the former so arranged that they can be lowered from the top, or raised from the bottom, and the light admitted from the top or bottom, or both at the same time, if desired. The white curtains raise and lower in the ordinary manner. This affords the best light I have ever had, and enables me to see into pulp cavities, and find root canals with greater ease than any of the former arrangements. Especially is this the case in operations upon the lower teeth. By shading the lower half of the window, the light admitted from the top only can be reflected into the pulp cavities with a concave mirror, enabling one to see distinctly where it would be impossible with the light admitted at the lower half of the window. Perhaps the best instruments yet placed in the market for removing the pulps of teeth are the broaches with a single hook at the point. The temper of the steel bristles is certainly excellent, but have never found any with a point well adapted to the purpose. The barbed broaches are only useful in large straight canals. They lack toughness of temper, the barbs are too long, and too far from the point. They can be improved by cutting off the points at the first barb, filing down to a small size, leaving only two barbs nearest the point, and these very short. I think if the steel bristles had two or three very short barbs near the point they would be superior to any instrument yet devised for pulp extirpation. Great care is necessary in these operations to avoid the danger of forcing any of the pulp debris, or the point of the instrument, or both, through the apical foramen. These nerve bristles referred to should be used very cautiously, as their small size, smooth round surface, and excellent spring temper, render them well adapted to follow small curved canals, and they pass through the foramina so easily in many cases that the membrane is wounded before we are aware of it. If any one doubts this, let him take a recently extracted tooth, and try the experiments, and he will find that in many of the buccal roots of tbe upper molars, even, these instruments will readily pass through the foramina half an inch or more. How much more easily, then, will they pass through the palatine roots of the same teeth, and also the large straight canals of the incisors and cuspids. As accidents of this kind always produce more or less irritation, and as prevention is better than cure, it is well to explore root canals carefully. In many cases considerable advantage is gained by reaming out the canals, especially where the pulps can be removed only in fragments. A reamer will scrape off the particles adhering to the walls of the canals, which a broach or hook will not do. Glidden's reamers, while not all that could be desired, are very useful instruments, and have aided me many times. The reamers recently devised by Dr. Talbot are also very valuable, and although I have not been able to accomplish with them all that the inventor claims they will do, yet would not like to be without them. The canals in the incisors, cuspids, and bicuspids, are not usually difficult to cleanse or fill. Neither are those in the palatine roots of the superior, and the distal roots of the inferior molars. The buccal roots of the upper molars being smaller, and usually somewhat flattened and curved, are often difficult to treat, and sometimes even to find. The mesial roots of the lower molars are also flattened, and the canals usually more or less contracted, resembling an hour glass in shape —this contraction frequently resulting in two distinct canals. When this is the case considerable difficulty is experienced in following them their entire length, and perhaps in some cases it is impossible to do so. The right and left exploring instruments, with long, fine points, are valuable for finding these small canals. Frequently the canals in the buccal roots of the upper molars, are contracted near the pulp chamber, and if they are reamed out at this point, can be easily followed their entire length. For this purpose a three-sided broach, with rather a soft spring temper and a small round point, is often sufficient. Any reamer for this location must be short enough to allow its entire length between the open jaws, that the instrument may be used in the direction of the canals. Glidden's reamers may also be used in the same wax by cutting off to the desired length and moulding some seating wav or modeling compound around the shaft for a handle. The canals should be thoroughly syringed with warm salt water after the pulps are removed. This is best done with the rubber dam still in position. After drying the cavities with spunk, I use fine tissue paper cut in triangular pieces and rolled into fine points for drying the canals, principally. These are easily used in all except the small contracted canals. The process is completed with the warm air cavity drier.

I do not usually fill the roots at the same sitting that the pulps are removed. Consider it safer practice to wait a few days, as some irritation may result from severing the pulp vessels, or some pulp debris may have been forced through the foramen. Prefer, therefore, to leave some cotton in the roots, moistened with carbolic acid or creosote, to render it antiseptic, and fill temporarily with gutta-percha. Neither do I regard it good practice to leave a cavity open after extirpating the pulp, as the food and fluids of the mouth soon find their way into the canals, where they decompose, often causing pericementitis, and in any case making it necesaary to do the work of cleansing all over again. For temporary fillings I use the common red gutta-percha, with the addition of enough pumice stone to render it sufficiently hard and brittle to manipulate easily. It is much less expensive than the white gutta-percha and answers the ordinary requirements of a temporary filling. I fill roots with the white gutta-percha, rolled out to the desired size with a warm knife. It is often necessary to cut off the extreme points to prevent carrying it through the foramen. For the small tortuous canals I use a solution of white gutta-percha in chloroform. This, I find by experi ments out of the mouth, can be carried with a fine bristle into suprisingly small canals. For some years I used this solution quite extensively, but the time required for hardening, the shrinkage, and the ease with which it is carried through the foramina in ordinary canals, are objections which do not exist in the use of solid gutta-percha. Considerable difference of opinion exists as to the necessity of filling these small canals at all—my own opinion being that it is by far the safer way to fill all canals that can possbly be filled, even though they require reaming and enlarging for the purpose. Have many times removed fillings from abscessed teeth and found the larger canals filled, but the smaller ones left untouched. The fact that cleansing, treating and filling these, resulted in a complete cure, is some evidence at least that the neglected canals were the cause of the trouble. As an experiment, I have frequently filled the larger canals, leaving the very small ones to take care of themselves, filling the cavities temporarily, and after a few weeks removing the filling, and the offensive odor, which I have invariably found to be present, is sufficient evidence to my mind that such is not safe practice. Have often been asked if I fill three canals in the upper molars. Will say that I do almost invariably. Indeed, do not recall a single case of a first upper molar in which I have failed to find and fill three canals, for at least eight or ten years, and but two instances of failure to find the third canal in the second upper molar, and perhaps three or four in the third, during the same period. I do not claim that these have *all* been filled *thoroughly* their entire length. This, as we all know, would in many cases be an impossibility; but have approximated thoroughness, as far as patience, perseverance, and my limited ability could do it. Am decidedly of the opinion, that if the profession generally felt that they were to receive a fee for these operations, commensurate with the time and labor necessary to be expended, greater thoroughness would prevail, and consequently more uniformly successful results be attained. The theory, that if the *end* of the canal is perfectly sealed, the balance may be left unfilled, while theoretically correct, is, I think, objectionable in practice, as we can seldom, if ever, *know* that the foramen *is* entirely closed. If it *does* leak, the open canal becomes a reservoir for the accumulation of moisture, which soon decomposes, and the gases thus generated escaping through the leaky plug, become a source of irritation to the surrounding membrane.

As previously stated, the treatment of an alveolar abscess depends upon the cause, and the condition in which we find it. If in the earlier stages of inflammation, and the cause is a dead or putrescent pulp, the thorough removal of the cause, with antiseptics left in the roots, and aconite and iodine applied to the gums, relief will generally soon follow, and an abscess be prevented. Among the various means resorted to for the purpose of preventing suppuration, are leeches applied to the gums, hot foot baths and cathartics, often with beneficial results. I am of the opinion, however, that where the inflammatory symptoms are sufficiently severe to require constitutional treatment, aconite and belladonna will usually afford all the relief that can be derived from the use of cathartic medicines. The heat, swelling, redness of the parts, the hot, dry skin, full and bounding pulse, pain in the head, and thirst, which are usually present in such cases, are symptoms more readily controlled by aconite and belladonna than by any other remedies I know of. I prefer either Fleming's tincture of aconite or the German tincture. Of the former, I generally use half a drop at a dose ; of the latter, one drop. The belladonna I prefer in the second or third decimal attenuation, in one or two drop doses. These, in severe cases, can be given in alternation every half hour for two or three hours, when the interval can be lengthened to one hour, and, as the inflammatory symptoms subside, to two hours. The common tincture of belladonna, usually kept in drug stores, is unreliable. The best I have ever used is the German tincture, obtained from the homœopathic pharmacies. The local remedies principally used in the treatment of alveolar abscesses are creosote, carbolic acid, iodine, salicylic acid, and the aromatic sulphuric acid.

The treatment after the cause is removed consists in bringing the remedy in contact with the pyogenic sac, cauterizing its entire surface, to break it up and promote healthy granulations. This is accomplished by injecting through the fistula, where one exists, with a Farrar's syringe, or by pumping the medicine through the root canal, where it is possible, using cotton wound on a broach for a piston. It often happens, however, that no fistula has been formed, and it is impossible to force the remedy through the canal. It has been recommended, in such cases, to drill through the root. This, in perfectly straight roots, if carefully done, is not bad practice; but, as we can never know positively that a root has not some curve, such a course is always risky. It is a little over eight years since I abandoned this practice; at that time two teeth were lost from this cause—an upper bicuspid and lateral incisor. The roots of both these teeth having an abrupt curve near their apices, my drill came out at the sides. I then decided to try cleansing the canals, treating with antiseptics, and trusting to nature for a cure.

When I state that no abscessed tooth has been lost since that time, where an opportunity has been afforded for carrying out my treatment, it will be inferred that the change has been a satisfactory one. The remedies which have given me the best results are the crosote of commerce (probably oily carbolic acid and creosote) and iodine. These I combine by dissolving the iodine in the creosote, leaving an excess of iodine.

After cleansing and drying as thoroughly as possible, I swab the canals with the remedy, using the cotton wound on a broach for the purpose, leaving cotton in the canals saturated with the medicine, and sealing with a temporary filling. If the liquid is carried through the foramen, as is often the case, a burning sensation will be experienced.

If the *liquid* does not go through, the vapors of the iodine will, if the cavity is perfectly sealed, unless the root is absolutely stopped. I treat these cases upon the hypothesis that where there is sufficient opening through the canal for the gasses from a decomposing pulp to escape, the vapors of iodine will find an exit—am not certain but the vapors coming in contact with the sac are quite as efficacious as the liquid, though where I *know* there is pus, I am not careful to prevent the medicine from going through in liquid form. On the other hand, where I doubt the existence of pus, I *am* careful to prevent the liquid from passing through the root, as an escharotic applied to the already inflamed membrane would likely result in suppuration. The vapors, instead of being escharotic, act as antiseptics and gentle stimulants to the membrane and weakened coats of the vessels. As an evidence that the iodine does find its way through the roots in some form, I will mention the fact that in nearly every case where a fistula exists, the patient complains of the taste of iodine until the fistula heals, although the cavity is filled as perfectly as I can fill it. Thinking perhaps my gutta-percha fillings leaked, I have filled, in some cases, with cement, and the taste of iodine continued. Where no fistula exists, soreness some times results from stopping up the cavity, and the patient should be instructed to return at once, or remove the temporary filling, if nesessary. The cases in which trouble is most likely to occur are nervous debilitated patients, confined by indoor employments. The operator should be governed by the condition of the patient at the time of treatment, whether it will be safe to seal up the cavity or not. The menstrual period, in delicate ladies suffering from uterine diseases, and the first months of pregnancy, are conditions requiring extreme caution.

I find my experiments out of the mouth, that iodine will go through the roots where carbolic acid will not. This was tested by winding the roots with cotton, and imbedding all but the crowns in plaster. I am aware that the conditions are not the same as the mouth, but the fact that the odor of the iodine was found upon the cotton where no trace of the acid could be discovered, is evidence that the vapors of the iodine found an exit through the apical foramina, where the acid did not. The principal objection urged against the use of iodine is that it discolors the teeth. Alcohol and carbolic acid have been used to wash it out. This they do by dissolving it, but at the same time it penetrates the dentine, leaving a slight stain. Aqua ammonia possesses the property of neutralizing the color of iodine; therefore, if the cavity is immediately washed with it, no stain remains. A colorless tincture of iodine is made by adding about one drachm of ammonia to one ounce of the tincture. In this proportion, the color disappears in about forty-eight hours. I have been using this, to a limited extent, for a few months, and I think it will prove a useful remedy. It certainly seems to arrest the secretion of pus promptly. For injecting a sac through the fistula, I know of nothing better than an alcoholic solution of salicylic acid. This, if the root is properly cleansed and treated, and the patient is in fair health, will usually effect a cure. In those cases with large foramina, where chronic inflammation remains after the secretion of pus has been arrested, considerable benefit may be derived from the use of the tincture of hydrastis, calendula, or sulphate of zinc and sulphate of morphia, each five grains to the ounce. The cotton in the canals should be changed frequently during treatment, as, if left too long, it becomes offensive, causing irritation to the already weakened membrane. External poultices should never be used in the treatment of this disease, neither should an abscess be allowed to break upon the face. Where poulticing is necessary, a roasted fig applied to the gum, is sufficient. I stated that no abscessed tooth had been lost during the past eight years where an opportunity had been afforded me for carrying out my treatment. While this is true, I wish to state distinctly that I have no idea that I can treat every case of this disease successfully. That I have been fortunate, I admit; but may fail in the very next case. I mention the fact reluctantly, and solely for the purpose of encouraging others to greater thoroughness and perseverance. I have two chronic cases under treatment at the present time which have given me considerable trouble, but both are nearly well, and I hope to save them. Will mention a case or two, which may be taken as an average of my cases and their treatment.

Case 1st. June, '76.—Mrs. I., abscess right superior first molar. Tooth sore, face and gums swollen and very painful; had been under treatment for weeks. She had been told it would require from six weeks to three months to effect a cure, with doubt expressed as to the result. Applied rubber dam, carefully excavating the cavity, and found that an attempt had been made to extirpate the pulp, through a small opening in the pulp chamber, which was so far from being successful that the buccal canals had scarcely been touched. Spent half a day of diligent labor in cleansing these canals; syringed, dried, and treated with the iodine and creosote, as before described, sealing up the cavity as usual. Painted the gums with aconite and iodine and dismissed the patient. In a few days the tooth was nearly well. I treated as before, and in three or four days filled the roots. In one week, filled the crown with gold. Last winter saw the case, and found it had given no trouble.

Case 2d. April, '81.—Mrs. C., from an adjoining town, abscess of left inferior first molar, with pus discharging through fistula; also, pericementitis of right inferior first bicuspid. Was half a day cleansing these canals; treated and filled, temporarily, as described; came again in one week, and both teeth, apparently, perfectly well. Filled root in bicuspid, and changed cotton in molar, not having time, between trains, to fill the roots.

Occasionally chronic abscesses, occurring in debilitated patients, require something more than local treatment. It is well known that if an ulcer or canker patch upon the lip or cheek is cauterized while the patient is debilitated, it is made worse; but if a tonic is administered for a few days previously, one application of the caustic is sufficient for a cure. The same is true in the treatment of abscesses. This is illustrated in the following cases: A colored girl, with abscess of the antrum, was treated locally for some weeks, and at times it appeared nearly well, but would again suppurate. A tonic of quinine and iron was prescribed, and the local treatment continued as before. From this time there was a marked improvement, which continued until a permanent cure was effected. A lady about seventy years of age, having worn an entire artificial denture for twenty years, was suffering from an abscess of the anterior portion of the superior maxilla, which had been treated by her physician for some months, local treatment being continued after coming under my care. This like the former case, was cured only after the administration of a tonic. One more case I desire to mention, is that of a lady recently recovered from an attack of intermittent fever, and also debilitated by nursing a large healthy boy the second summer. An abscess of the left superior lateral incisor resisted all local treatment until a tonic was administered, after which one application of salicylic acid to the sac through the fistula effected a complete cure.

I have omitted, but wish here to state, that the only certain test I have ever found for the vitality of a pulp is the rigolene spray applied to the tooth after it has been isolated by the adjustment of the rubber dam.

In conclusion, I will say that, aside from necrotic conditions, I think failures result principally from four causes, viz: imperfect cleansing of the pulp chambers and root canals, forcing foreign matter through the foramina, over medication, or the too frequent application of caustic remedies, keeping up the secretion of pus; leaving cotton too long in the roots until it becomes offensive, causing irritation; and finally, by not paying sufficient attention to the general health of the patients.—*Transactions Illinois State Dental Society*, 1881.

